# Personality Disorders in Childhood: Validity of the Coolidge Personality and Neuropsychological Inventory for Children (CPNI)

**DOI:** 10.3390/ijerph19074050

**Published:** 2022-03-29

**Authors:** Alexandro Fortunato, Annalisa Tanzilli, Vittorio Lingiardi, Anna Maria Speranza

**Affiliations:** Department of Dynamic and Clinical Psychology, and Health Studies, “Sapienza”, University of Rome, Via degli Apuli 1, 00185 Rome, Italy; annalisa.tanzilli@uniroma1.it (A.T.); vittorio.lingiardi@uniroma1.it (V.L.); annamaria.speranza@uniroma1.it (A.M.S.)

**Keywords:** assessment, childhood personality, diagnosis, personality disorder, CPNI

## Abstract

Background: A growing body of evidence has shown that maladaptive traits and emerging patterns of personality can be traced to an early stage of development and may be assessed in childhood. The goal of present study was to provide preliminary data on the validity of the Coolidge Personality and Neuropsychological Inventory for Children (CPNI), an instrument designed to assess personality pathologies and other clinical conditions in childhood. Method: A sample of 146 clinicians completed the CPNI, as well as the Child Behavior Checklist (CBCL) to evaluate the behavioral problems and social competencies, regarding a child (aged 6–11 years) who had been in their care between 2 and 12 months. The clinicians also filled out a clinical questionnaire to provide information on the children, their families, and psychotherapies. Results: There were significant and clinically consistent associations between the CPNI and CBCL. They confirmed the good concurrent (convergent and discriminant) validity of the CPNI. Conclusions: The findings seem to support the validity of the CPNI as diagnostic instrument, taking children’s PDs and behavioral problems into account. Despite some limitations, the CPNI represents a helpful measure to evaluate the children’s personality configurations according to the DSM model. It may be employed along with other tools based on other diagnostic frameworks within the context of a multi-method and multi-informant assessment to provide an accurate and comprehensive formulation of children’s overall functioning.

## 1. Introduction

A growing literature on emerging personality pathologies in childhood (for a review, see [1]) has shown that maladaptive personality traits and patterns can be traced to an early stage of development and, accordingly, may be assessed in childhood [2,3,4,5,6]. Distinct ways of thinking, feeling, and behaving take shape in childhood from an interaction of genetic and psychosocial factors, and tend to persist over time [7]. Evidence has demonstrated that these emerging personality patterns organize and structure the quality of children’s inner experience and impact defensive and coping strategies, relatedness, cognitive processes, and adaptation [8,9,10,11,12,13,14,15]. However, some central questions in this field remain open: How should these dysfunctional traits and patterns be investigated in children, considering that development makes the investigation of their mental functioning and personality more challenging? Is it better to let development run its course, with the risk of pathologizing, or to make a PD diagnosis in childhood and consequently intervene to prevent maladaptive personality patterns from becoming stable and exacerbated in adolescence and adulthood? According to the Psychodynamic Diagnostic Manual-Second Edition (PDM-2) [12], personality pathologies in childhood and adolescence are distinct from both normal development and abnormal psychopathology; moreover, they should be considered as evolving, unfixed, and dynamic. It is crucial to promote an accurate assessment of the core personality characteristics of children to better understand their psychological functioning and plan individualized preventive interventions.

Empirical studies and clinical observations seem to support that some PDs arise with the same features in childhood as in adulthood. Given that these psychopathological characteristics are not reducible to other psychiatric conditions, they require clinical attention [3,16,17,18]. Children rarely receive a specific diagnosis of personality; however, they do suffer from these emerging pathologies, often remaining positioned in a shadow zone. Evaluating the children’s personalities is critical in clinical practice. It enables investigating the risk and protective factors and the developmental trajectories of these pathologies, as well as to have more complete knowledge of the mental functioning of children, and to better identify how (potentially comorbid) clinical conditions manifest in the context of mental functioning and personality in childhood [7,11,12]. For example, some studies have highlighted the relationships between specific PDs and several psychiatric syndromes, such as the link between the borderline personality and attention deficit hyperactivity disorder (ADHD), dysphoric personality and depressive/anxious disorders, or obsessive personality and obsessive-compulsive disorder e.g., [2]. Moreover, some children with an autism diagnosis fall into the schizoid personality [2].

According to the Diagnostic and Statistical Manual of Mental Disorders in its latest editions (DSM-IV and DSM-5) [19,20], a diagnosis of PD can be made for patients aged 18 years and older. Nonetheless, strong markers of a disorder can be observed in younger patients after careful clinical observation and examination. The Coolidge Personality and Neuropsychological Inventory for Children (CPNI) [21] was designed to assess PDs in children and adolescents, using DSM-IV and DSM-5 criteria [19,20]. The measure also covers other clinical conditions—such as depression, anxiety, and eating disorders—as well as neuropsychological and neurocognitive disorders and executive function problems. Therefore, it assesses children and adolescents at risk for numerous psycho(patho)logical syndromes. In more detail, the CPNI consists of the following scales: (a) a validity scale that verifies the tendency to deny pathology; (b) 12 PD scales based on DSM-IV and DSM-5 approaches [19,20] (such as paranoid, borderline, dependent, histrionic, obsessive-compulsive, schizotypal, schizoid, narcissistic, conduct disorder or antisocial, avoidant, passive-aggressive, and depressive), and five scales reporting personality changes due to medical conditions; (c) seven major clinical disorders (such as general anxiety disorder, depression, separation anxiety disorder, oppositional defiant disorder, gender dysphoria, anorexia nervosa, and bulimia nervosa); (d) six neuropsychological disorder scales (such as ADHD inattention subtype, ADHD hyperactivity-impulsivity subtype and ADHD combined, mild neurocognitive disorder, postconcussional disorder and executive dysfunction of the frontal lobes) and three subscales regarding executive dysfunction; (e) one neuropsychological scale regarding neuropsychological dysfunction and 12 subscales; (f) eleven other clinical scales (such as emotional lability, disinhibition, aggression, apathy, paranoia, psychotic thinking, emotional coldness, sleep disturbances, social anxiety, social withdrawal, and self-esteem); (g) four hostility scales (such as antisocial triumvirate, dangerousness, conduct disorder–aggressive subtype, conduct disorder–delinquent subtype).

The CPNI uses a dimensional rating approach, with cut-off scores established at one and two standard deviations, T-scores (standard scores with a mean of 50 and a standard deviation of 10), and percentiles. The normative sample was composed of 780 American children (390 female and 390 male) who ranged in age from 5 to 17 years. It was used to study the features and correlates of specific PDs [22,23], to identify the features of specific populations [24,25,26], and to compare different models of PD assessment [27].

The CPNI uses a parent or teacher as an informant. Parent reports may be affected by a lack of insight, implicit defense mechanisms, social desirability bias, or an inability to see the child’s suffering due to their own. Furthermore, although teachers spend many hours with children and know them well, they are not sufficiently trained to assess personality and can suffer from the same parental biases. Rather, research has shown that clinicians represent the most trustworthy informants, with respect to personality [2,28,29].

The goal of the present study was to provide preliminary data on the validity of the CPNI in an Italian sample according to the clinician’s perspective. To the best of our knowledge, the CPNI had never been administered in Italy, prior to our research. Consistent with the clinical and empirical literature [22,23,24,25,26,27], it is possible to hypothesize that the CPNI would represent a valid assessment tool with a good concurrent (convergent and discriminant) validity verified through associations between the CPNI and CBCL.

## 2. Materials and Methods

### 2.1. Participant Sampling

An Italian sample of experienced clinicians was recruited from the membership rosters of national associations of developmental psychotherapy and centers specialized in the treatment of children, using a practice network approach. Clinicians had at least 3 years of post-psychotherapy licensure experience and treated children for at least 10 h per week. They agreed to participate in a study on psychological assessment in childhood and collected data of the children in their care, without patient involvement.

Clinicians were directed to select one child patient in their caseload according to the following inclusion/exclusion criteria: (a) aged 6–11 years; (b) no psychotic psychiatric disorder based on the DSM–5 [20] classification system; (c) not receiving drug therapy for psychotic symptoms; (d) no traumatic brain injury, neurological disorder, and/or clinically significant cognitive impairment; (e) no autistic spectrum disorder; and (f) in treatment between 2 and 12 months. The children do not necessarily have a psychiatric diagnosis. Clinicians had to describe a child patient who has enduring patterns of thoughts, feelings, or behaviors—that is, personality problems—that cause distress or dysfunction. To minimize patient selection bias, clinicians were asked to provide data on the last patient they saw in the previous week who met the study criteria. All clinicians received no compensation.

### 2.2. Clinician Characteristics

The sample comprised 146 clinicians, of whom 124 (85%) were women and 22 (15%) were men. Clinicians’ principal theoretical and clinical approaches included psychodynamic/psychoanalytic (*n* = 82), cognitive/behavioral (*n* = 34), systemic/relational (*n* = 8), integrated (*n* = 17), and other (*n* = 5). Average length of clinical experience was 10.97 years (*SD* = 7.04; range = 3–33) and average length of treatment was 7.45 months (*SD* = 3.65; range = 2–12).

### 2.3. Patient Characteristics

The sample included 146 patients, of whom 74 (50.7%) were female and 72 (49.3%) were male. Children’s average age was 8.9 years (*SD* = 1.6; range 6–11). Amongst them, 107 (73.3%) had a DSM-5 [20] psychiatric diagnosis, including specific learning disorder (*n* = 20, 18.69%); attention-deficit/hyperactivity disorder (*n* = 18, 16.82%); depressive disorder (*n* = 15, 14.02%); anxiety disorder (*n* = 13, 12.15%); disruptive, impulse control, and conduct disorders (*n* = 10, 9.35%); communication disorder (*n* = 9, 8.41%); obsessive-compulsive disorder (*n* = 8, 7.48%); and post-traumatic stress disorder (*n* = 6, 5.61%). The remaining 7.47% were diagnosed with sleep–wake disorder, relational disorder, feeding disorder, attachment disorder, evacuation disorder, and/or motor disorder.

### 2.4. Measures

Clinical questionnaire: We designed an ad hoc questionnaire for clinicians to report general information about themselves, their patients, and their therapies [2,11]. Specifically, clinicians provided basic demographic data, referring to their age, gender, race, profession (i.e., psychiatrist or psychologist), years of experience, and theoretical orientation. They also provided information on their patients’ demographic, diagnostic, developmental, and family history, citing the presence of any traumatic experiences or events (e.g., neglect or mistreatment, parental abandonment, early separation) and indicating the length of treatment.

Coolidge Personality and Neuropsychological Inventory for Children: The CPNI [21] is a 200-item pencil-and-paper test designed to be filled out by the parent or guardian of the child or adolescent subject, or someone (e.g., a teacher) who is intimately familiar with the subject. The first 198 items are answered on a 4-point Likert-type scale ranging from 1 (strongly false) to 4 (strongly true). Items 199 and 200 are answered either True or False. The scale takes 30–45 min to complete, and it is designed for assessing children aged 5–17 years. It contains 1 validity scale and 49 clinical scales to evaluate different aspects of childhood functioning: (1) personality, (2) neuropsychological problems, (3) clinical diagnoses, and (4) various aspects of child functioning. The measure is compiled by a clinician. Prior to the present research, there was no Italian version. Thus, we translated it into Italian and a native English speaker translated it back into English to verify the accuracy of the translation.

Child Behavior Checklist–Clinician Version: The CBCL [30] is a clinician-report measure of children’s behavioral and emotional difficulties and social competencies, which is used to investigate a broad spectrum of developmental characteristics in children and adolescents. The measure evaluates behavior using two ‘broad band’ scales, referring to internalizing and externalizing symptomatology, respectively. The entire checklist comprises 128 items, which are grouped into 11 problem scales and 4 competence scales. The CBCL has been shown to have high levels of validity and reliability, similar to those of the parent- and teacher-report versions [30,31].

### 2.5. Statistical Analysis

Statistical analyses were performed using SPSS 24 for Windows (IBM, Armonk, NY, USA). Descriptive analyses were performed to verify the presence of PDs in the Italian sample of children. Bivariate correlations (Pearson’s *r*, two-tailed) between the CPNI personality scales and the CBCL symptomatology scales were carried out to investigate the concurrent (criterion) validity of the CPNI.

To ensure a thorough and psychometrically robust exploration of the convergent and discriminant validity of this use of the CPNI, a series of stepwise multiple regression analyses were performed to identify which specific dimensions of the CPNI predicted distinct symptomatology patterns and psychopathological problems identified with the CBCL. In these regression analyses (in which symptomatology variables were used as criterion variables), all CPNI personality scales were entered as potential predictors. Change in *R*^2^ was used to determine the predictive power of each variable. The F test (i.e., *F*-change) was used to determine whether a change in *R^2^* was statistically significant (at *p* ≤ 0.05).

## 3. Results

Table 1 presents the diagnoses that emerged from the standardized scores of the CPNI PD scales. Forty-four children (30.1%) had a single PD diagnosis, while 51 (35%) had more than one diagnosis. Thus, in total, 95 (65.1%) children were diagnosed with at least one PD. In the remaining 51 (34.9%) children, no PD was diagnosed.

Table 2 shows the comorbidity between PDs. Although half of the sample had one to three diagnoses, some patients had up to seven comorbid diagnoses.

To examine the criterion validity of the CPNI, we investigated the relationships between children’s PDs (as assessed by the CPNI) and a wide range of symptoms and dysfunctional behaviors (as assessed by the CBCL). In more detail, the 12 PD scales of the CPNI correlated with all CBCL scales. Table 3 depicts the results of the correlation analyses, which demonstrated good concurrent validity.

Overall, all PD scales were strongly and positively associated with CBCL total problems. Moreover, the PD scales were significantly associated with CBCL scales in a clinically coherent and psychometrically robust way. Of note, internalizing problems were significantly and strongly associated with obsessive-compulsive, dependent, schizotypal, schizoid, avoidant, and depressive personalities; whereas externalizing problems were significantly and strongly associated with paranoid, borderline, narcissistic, histrionic, and passive-aggressive personalities.

Two stepwise multiple regressions were performed to test the CPNI’s validity at a more specific level of analysis. Of note, distinct CPNI PD scales were identified as predicting children’s behavioral and emotional difficulties, and social problems. Overall, some PD scales predicted distinct symptom patterns, supporting good convergent and discriminant validity of the CPNI (Table 4). In particular, internalizing problems were predicted by depressive and avoidant personalities, while externalizing problems were predicted by antisocial, passive-aggressive, and borderline personalities. The most compromised personality diagnoses that predicted total problems were borderline, schizotypal, antisocial, passive-aggressive, and paranoid.

## 4. Discussion

The present study aimed at providing the preliminary data on psychometric properties (validity) of the CPNI’s clinician version in assessing maladaptive personality traits and patterns in childhood. The results confirmed that the CPNI has a good concurrent validity due to the strong associations between its PD scales and CBCL psychopathological dimensions. 

The research showed that more than 65% of the children evaluated with the CPNI were diagnosed with at least one PD. Notably, this finding must be interpreted in light of the sample which is composed of children who were all in evaluation or treatment [32]; therefore, all of the children presented some issues although not all of them had a clinical diagnosis. Nonetheless, this is a clinically relevant result in the context of the literature on PDs [2,7,11,12,33,34,35]. 

Overall, the CPNI appeared to present good convergent and discriminant validity. Looking at the results depicted in Table 3, it is important to highlight the significantly robust and clinically coherent correlations between the CPNI PD scales and several psychopathological problems (evaluated using the CBCL) in children. Notably, internalizing problems were related to obsessive-compulsive, dependent, schizotypal, schizoid, avoidant, and depressive personalities [36,37]. Likewise, externalizing problems were strongly associated with paranoid, borderline, narcissistic, histrionic, and passive-aggressive personalities [36,38]. The positive associations found between all PD scales and total problems are particularly meaningful for their clinical implications and the planning of effective treatments in childhood [7,11].

More in detail, consistent with the literature, emerging paranoid personalities (relatively rare in childhood; [2,11]) that represent pervasive patterns of distrustfulness and hostility were mainly correlated with aggressive behavior and social problems [39]. On the contrary, schizoid and schizotypal personalities were correlated with withdrawal, as well as social, thought, and attention problems [40], probably due to the children’s severe detachment from interpersonal relationships, their very restricted range of expressions and emotions, along with the presence of unconventional beliefs and odd behaviors.

Borderline and histrionic personalities that are distinguished by an impaired ability to regulate the emotions, and the tendency to act on impulses, showed a significant and positive correlation with externalizing problems [2], that were also related to antisocial [41], narcissistic [42,43], and passive-aggressive personalities [44,45], taking into account the attitude of these patients to be angry or hostile, self-centered, and lacking in empathy. In line with clinical observations and empirical research, externalizing problems were also significantly and positively correlated with social, thought, and attention problems, and delinquent and aggressive behavior [46,47]. 

Consistent with the literature [1], obsessive-compulsive personalities that are characterized by a generalized preoccupation with orderliness, perfectionism, and mental and interpersonal control—at the expense of flexibility, openness, and efficiency [11,12]—were associated with anxiety/depression and social and thought problems. On the contrary, dependent and avoidant personalities revealed a high correlation with anxiety/depression and withdrawal, as well as social, thought, and attention problems [18,48,49,50]. Similarly, depressive personality was correlated with anxiety/depression, withdrawal, somatic complaints, and social problems [51]. Overall, according to the clinical and empirical research [2,7,11,12], these PDs—mostly related to internalizing problems—tended to experience chronic painful emotions, especially depression and anxiety, as well as being emotionally inhibited and socially avoidant.

Looking at Table 4, the present study revealed that some PDs were associated with distinct symptomatic patterns in predictable and clinically meaningful ways. Consistent with the diagnostic framework of the PDM-2 [12], these results seem to support the role of personality as the context for psychopathology. In fact, internalizing problems were predicted by depressive and avoidant personalities [12,18], while externalizing problems were predicted by antisocial, passive-aggressive, and borderline personalities [44,45,46,47,48]. Total problems were predicted by borderline, schizotypal, antisocial, passive-aggressive, and paranoid personalities [1,39,44,45], emphasizing that these personalities were the most compromised.

The other PDs that predicted symptomatic patterns were as follows:

- anxiety and depression were predicted by dependent and depressive personalities [48,51];
- withdrawal was mostly predicted by schizoid, schizotypal, and avoidant personalities [18,40];
- somatic complaints were predicted by depressive personality [51];
- social problems were mostly predicted by borderline and schizotypal personalities [40,46,47];
- thought problems were predicted by schizotypal personality [34];
- attention problems were predicted by borderline, schizotypal, passive-aggressive, paranoid, and antisocial personalities [22,40,41,44,45]; and
- delinquent and aggressive behaviors were predicted by antisocial and borderline personalities [41,46,47].

Consistent with the diagnostic framework of the PDM-2 [12], these findings—in addition to confirming the hypothesis—support the theoretical assumption which has strong clinical implications. Indeed, the presence of PDs represents a strong risk factor for development, and children who are diagnosed accordingly require an in-depth clinical assessment and appropriate clinical intervention to promote psychological health.

Although the CPNI is a useful and valid tool in evaluating personality pathologies in childhood, the comorbidity of PDs is high, and children may also receive six different diagnoses. The CPNI’s limitation is shared with other instruments based on the DSM diagnostic categories for PDs [28,29,52], and this finding may suggest a lack of discriminant validity of the constructs of PDs included in the international manual. 

For this reason, it seems crucial to integrate the use of CPNI based on the DSM models with tools such as the PDC-C [11] of the PMD-2 [12] or the CPAP-Q [2] to provide a deep and comprehensive formulation of a child’s overall functioning. These instruments do not represent an alternative to the CPNI but are complementary. They may promote a complete and clinically sensitive assessment able to capture variations in child functioning, even within the same diagnostic category, illuminate individual strengths and limitations, and plan a patient-tailored intervention.

## 5. Conclusions

The present study suggests that the CPNI is an appropriate and reliable tool for assessing childhood personality. The findings showed strong associations among all of the investigated variables, supporting the validity of this measure. Good preliminary evidence was also generated for the clinical utility of the CPNI, with respect to clinicians of various theoretical orientations. In detail, one strength of the CPNI is its ability to capture PDs in childhood; whereas a limitation is the low external validity that reflects the high comorbidities among different personalities.

Some limitations of the research design should be acknowledged. The first concerns the exclusive use of clinician-report instruments to obtain data about both the patients’ diagnoses. Future research should include multiple observers and tools to fully establish the diagnostic utility of the CPNI. Second, the sample is exclusively composed of children under evaluation or treatment although not all of them have a clinical diagnosis. Nonetheless, the CPNI promises to provide a helpful contribution in the field of personality assessment, especially combined with other instruments based on a more idiographic knowledge of patients. 

## Figures and Tables

**Table 1:** Frequency and Percentage of Personality Disorders (*N* = 146).

	Frequency	Percentage
BPD	4	2.7
OCPD	1	0.7
DPD	4	2.7
STPD	2	1.4
NPD	5	3.4
ASPD	1	0.7
SZPD	8	5.5
AVPD	12	8.2
HPD	1	0.7
PAPD	1	0.7
DEPD	5	3.4
Comorbidity	51	34.9
No diagnosis	51	34.9
Total	146	100

Note: BPD = borderline personality disorder; OCPD = obsessive-compulsive personality disorder; DPD = dependent personality disorder; STPD = schizotypal personality disorder; NPD = narcissistic personality disorder; ASPD = antisocial personality disorder; SZPD = schizoid personality disorder; AVPD = avoidant personality disorder; HPD = histrionic personality disorder; PAPD = passive-aggressive personality disorder; and DEPD = depressive personality disorder.

**Table 2:** Frequency and Percentage of Personality Disorders (*N* = 146).

	Frequency	Percentage
No diagnosis	51	34.9
1 diagnosis	44	30.1
2 diagnoses	14	9.6
3 diagnoses	12	8.2
4 diagnoses	11	7.5
5 diagnoses	8	5.5
6 diagnoses	2	1.4
7 diagnoses	4	2.7
Total	146	100

**Table 3:** Bivariate Correlations between CBCL ^a^ Behavioral and Emotional Difficulties and Social Problems and CPNI ^b^ Personality Disorders (*N* = 146).

	CBCL										
CPNI	Anxiety/ Depression	Withdrawal	Somatic Complaints	Social Problems	Thought Problems	Attention Problems	Delinquent Behavior	Aggressive Behavior	Internalizing	Externalizing	Total Problems
PPD	0.16	−0.92	−0.01	0.34 ***	0.02	0.18 *	0.26 **	0.49 ***	0.06	0.47 ***	0.36 ***
BPD	0.21 **	0.08	0.06	0.49 ***	0.27 ***	0.47 ***	0.47 ***	0.62 ***	0.12	0.58 ***	0.63 ***
OCPD	0.30 ***	0.12	0.09	0.19 *	0.17 *	0.01	−0.01	0.10	0.20 *	0.04	0.21 *
DPD	0.47 ***	0.22 **	0.16	0.40 ***	0.22 **	0.25 **	0.04	0.08	0.33 ***	0.06	0.38 ***
STPD	0.15	0.52 ***	0.06	0.44 ***	0.61 ***	0.31 ***	0.12	−0.06	0.22 **	−0.05	0.42 ***
NPD	0.12	0.03	−0.06	0.20 *	0.04	0.13	0.17 *	0.21 **	−0.01	0.21 **	0.24 **
ASPD	0.03	−0.05	0.02	0.27 ***	0.19 *	0.37 ***	0.61 ***	0.65 ***	−0.01	0.65 ***	0.51 ***
SZPD	0.10	0.56 ***	−0.05	0.27 ***	0.26 **	0.12	0.10	−0.04	0.26 **	0.02	0.27 ***
AVPD	0.42 ***	0.37 ***	0.10	0.31 ***	0.01	0.05	−0.08	−0.11	0.46 ***	−0.09	0.20 *
HPD	0.23 **	0.14	0.10	0.41 ***	0.22 **	0.35 ***	0.22 **	0.29 ***	0.14	0.23 **	0.45 ***
PAPD	0.13	0.11	0.01	0.40 ***	0.06	0.41 ***	0.34 ***	0.58 ***	0.11	0.53 ***	0.51 ***
DEPD	0.56 ***	0.21 **	0.20 *	0.20 *	0.09	0.03	−0.09	0.03	0.49 ***	0.04	0.24 **

^a^ Child Behavior Checklist–Clinician Version (CBCL; [26]). ^b^ Coolidge Personality and Neuropsychological Inventory for Children (CPNI; [17]). * *p* ≤ 0.05 ** *p* ≤ 0.01 *** *p* ≤ 0.001. PPD = paranoid personality disorder; BPD = borderline personality disorder; OCPD = obsessive-compulsive personality disorder; DPD = dependent personality disorder; STPD = schizotypal personality disorder; NPD = narcissistic personality disorder; ASPD = antisocial personality disorder; SZPD = schizoid personality disorder; AVPD = avoidant personality disorder; HPD = histrionic personality disorder; PAPD = passive-aggressive personality disorder; and DEPD = depressive personality disorder.

**Table 4:** Stepwise Multiple Regression Analyses Predicting Children’s CBCL ^a^ Behavioral and Emotional Difficulties and Social Problems, on the Basis of CPNI ^b^ Personality Disorders (*N* = 146).

	*R*	*R* ^2^	Standardized β	*F*-Change (Model)	*p*
Criterion variable: Anxiety/depression					
Step 1	0.56	0.32		66.99	<0.001
DPD			0.56		
Step 2	0.61	0.37		12.32	0.001
DPD			0.44		
DEPD			0.26		
Criterion variable: Withdrawal					
Step 1	0.56	0.32		66.39	<0.001
SZPD			0.56		
Step 2	0.62	0.39		16.91	<0.001
SZPD			0.40		
STPD			0.31		
Step 3	0.64	0.41		6.28	0.013
SZPD			0.36		
STPD			0.29		
AVPD			0.17		
Step 4	0.67	0.44		7.42	0.007
SZPD			0.33		
STPD			0.32		
AVPD			0.21		
PPD			−0.18		
Step 5	0.68	0.46		5.24	0.024
SZPD			0.31		
STPD			0.32		
AVPD			0.21		
PPD			−0.29		
PAPD			0.18		
Criterion variable: Somatic Complaints					
Step 1	0.20	0.04		5.89	0.016
DEPD			0.20		
Criterion variable: Social problems					
Step 1	0.49	0.24		44.90	<0.001
BPD			0.49		
Step 2	0.57	0.33		18.87	<0.001
BPD			0.39		
STPD			0.31		
Step 3	0.59	0.35		5.14	0.025
BPD			0.37		
STPD			0.27		
AVPD			0.16		
Criterion variable: Thought problems					
Step 1	0.61	0.38		87.20	<0.001
STPD			0.62		
Step 2	0.64	0.41		7.06	0.009
STPD			0.68		
AVPD			−0.18		
Criterion variable: Attention problems					
Step 1	0.47	0.22		39.73	<0.001
BPD			0.47		
Step 2	0.50	0.25		5.85	0.017
BPD			0.41		
STPD			0.19		
Step 3	0.52	0.27		5.24	0.024
BPD			0.45		
STPD			0.22		
OCPD			−0.17		
Step 4	0.56	0.31		7.26	0.008
BPD			0.27		
STPD			0.25		
OCPD			−0.22		
PAPD			0.26		
Step 5	0.59	0.34		7.05	0.009
BPD			0.38		
STPD			0.24		
OCPD			−0.19		
PAPD			0.35		
PPD			0.26		
Step 6	0.60	0.36		4.26	0.041
BPD			0.28		
STPD			0.25		
OCPD			−0.17		
PAPD			0.34		
PPD			0.27		
ASPD			0.17		
Criterion variable: Delinquent behavior					
Step 1	0.61	0.37		84.27	<0.001
ASPD			0.61		
Step 2	0.63	0.40		6.08	0.015
ASPD			0.50		
BPD			0.19		
Step 3	0.66	0.44		10.70	0.001
ASPD			0.45		
BPD			0.30		
DEPD			−0.23		
Criterion variable: Aggressive behavior					
Step 1	0.65	0.43		106.50	<0.001
ASPD			0.64		
Step 2	0.74	0.55		39.55	<0.001
ASPD			0.49		
PAPD			0.37		
Step 3	0.76	0.57		7.90	0.006
ASPD			0.50		
PAPD			0.39		
STPD			−0.17		
Step 4	0.78	0.61		12.36	0.001
ASPD			0.39		
PAPD			0.24		
STPD			−0.23		
BPD			0.31		
Step 5	0.79	0.63		8.05	0.005
ASPD			0.37		
PAPD			0.27		
STPD			−0.22		
BPD			0.37		
DEPD			−0.18		
Criterion variable: Internalizing					
Step 1	0.49	0.24		45.39	<0.001
DEPD			0.49		
Step 2	0.54	0.29		9.64	0.002
DEPD			0.34		
AVPD			0.27		
Step 3	0.56	0.31		4.18	0.043
DEPD			0.35		
AVPD			0.21		
SZPD			0.15		
Criterion variable: Externalizing					
Step 1	0.65	0.42		103.18	<0.001
ASPD			0.65		
Step 2	0.71	0.51		25.82	<0.001
ASPD			0.52		
PAPD			0.32		
Step 3	0.73	0.53		6.19	0.014
ASPD			0.53		
PAPD			0.34		
STPD			−0.15		
Step 4	0.74	0.55		8.08	0.005
ASPD			0.44		
PAPD			0.21		
STPD			−0.20		
BPD			0.25		
Step 5	0.75	0.57		4.13	0.044
ASPD			0.42		
PAPD			0.22		
STPD			−0.17		
BPD			0.30		
DEPD			−0.13		
Criterion variable: Total problems					
Step 1	0.63	0.40		95.11	<0.001
BPD			0.63		
Step 2	0.67	0.45		14.67	<0.001
BPD			0.55		
STPD			0.27		
Step 3	0.71	0.50		12.80	<0.001
BPD			0.41		
STPD			0.29		
ASPD			0.23		
Step 4	0.72	0.52		6.06	0.015
BPD			0.31		
STPD			0.30		
ASPD			0.23		
PAPD			0.16		
Step 5	0.73	0.53		4.00	0.047
BPD			0.37		
STPD			0.30		
ASPD			0.24		
PAPD			0.22		
PPD			0.17		

^a^ Child Behavior Checklist–Clinician Version (CBCL; [26]). ^b^ Coolidge Personality and Neuropsychological Inventory for Children (CPNI; [17]). DPD = dependent personality disorder; DEPD = depressive personality disorder; SZPD = schizoid personality disorder; STPD = schizotypal personality disorder; AVPD = avoidant personality disorder; PPD = paranoid personality disorder; PAPD = passive-aggressive personality disorder; BPD = borderline personality disorder; and ASPD = antisocial personality disorder.

## Data Availability

Not applicable.
